# Supplementary figures and images for: Transcranial direct current stimulation of cerebellum alters spiking precision in cerebellar cortex: A modeling study of cellular responses
Source: PLoS Comput Biol. 2021 Dec 9;17(12):e1009609. doi: 10.1371/journal.pcbi.1009609 (PMC8691604; doi:10.1371/journal.pcbi.1009609)

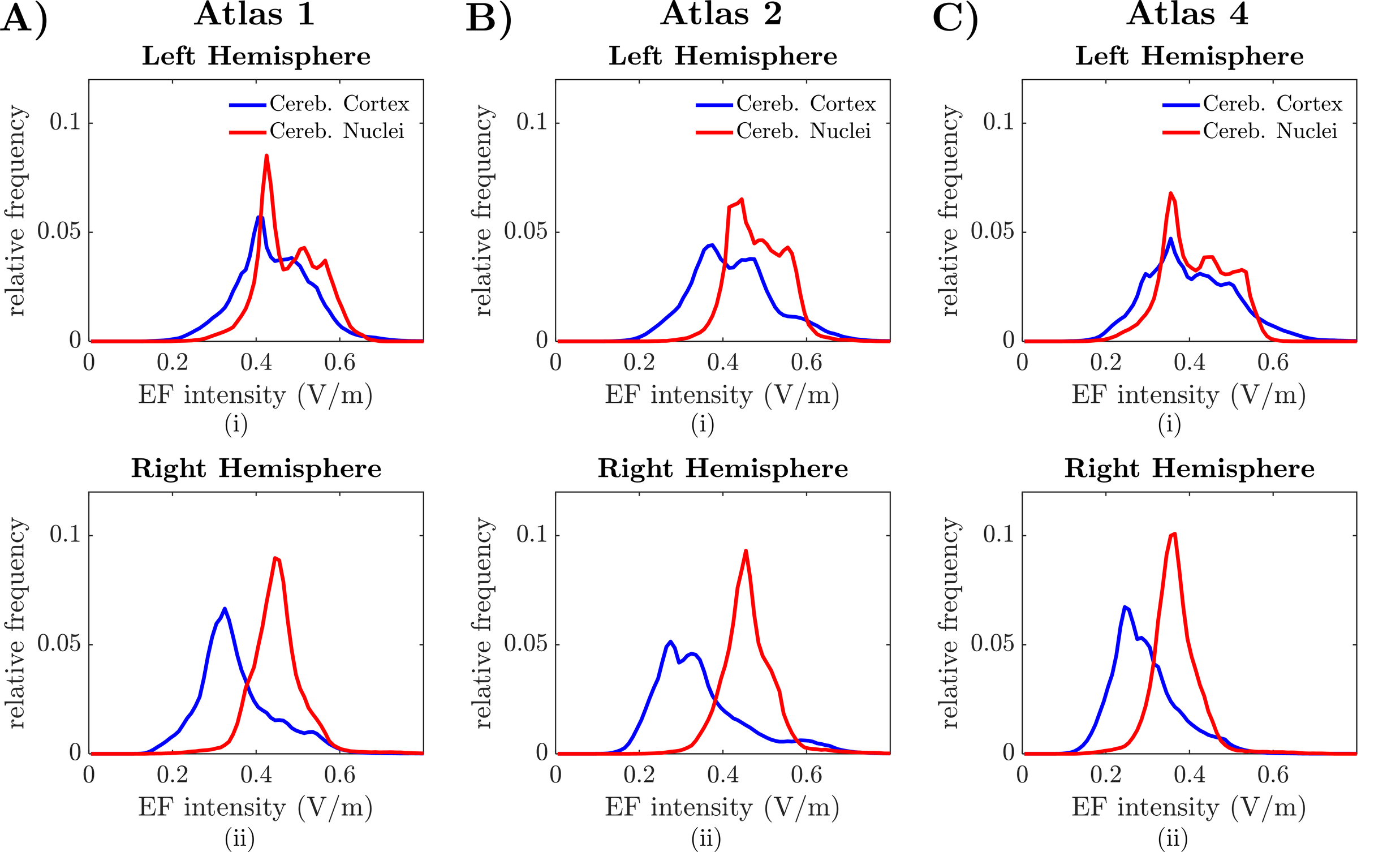

Supplement: S1 Fig — (A-C) Sample distribution of electric field (EF) intensity estimated for atlas 1 (A), 2 (B), and 4 (C) from [26], respectively, under regular (R)-tDCS with two pad electrodes. R-tDCS montage is as depicted in Fig 1A. Panels (i) and (ii) in (A-C) report the EF intensity distribution for the left (target) cerebellar hemisphere (i) and the right cerebellar hemisphere (ii), respectively. For each hemisphere and atlas, probability functions are estimated separately for voxels mapping the cerebellar cortex (blue lines) and the cerebellar nuclei (red lines). These EF intensity distribution functions complement the presentation of the results in Figs 1A and 2A. Sample probability distribution functions were computed as in Fig 2A. (TIF) [file pcbi.1009609.s001.tif]

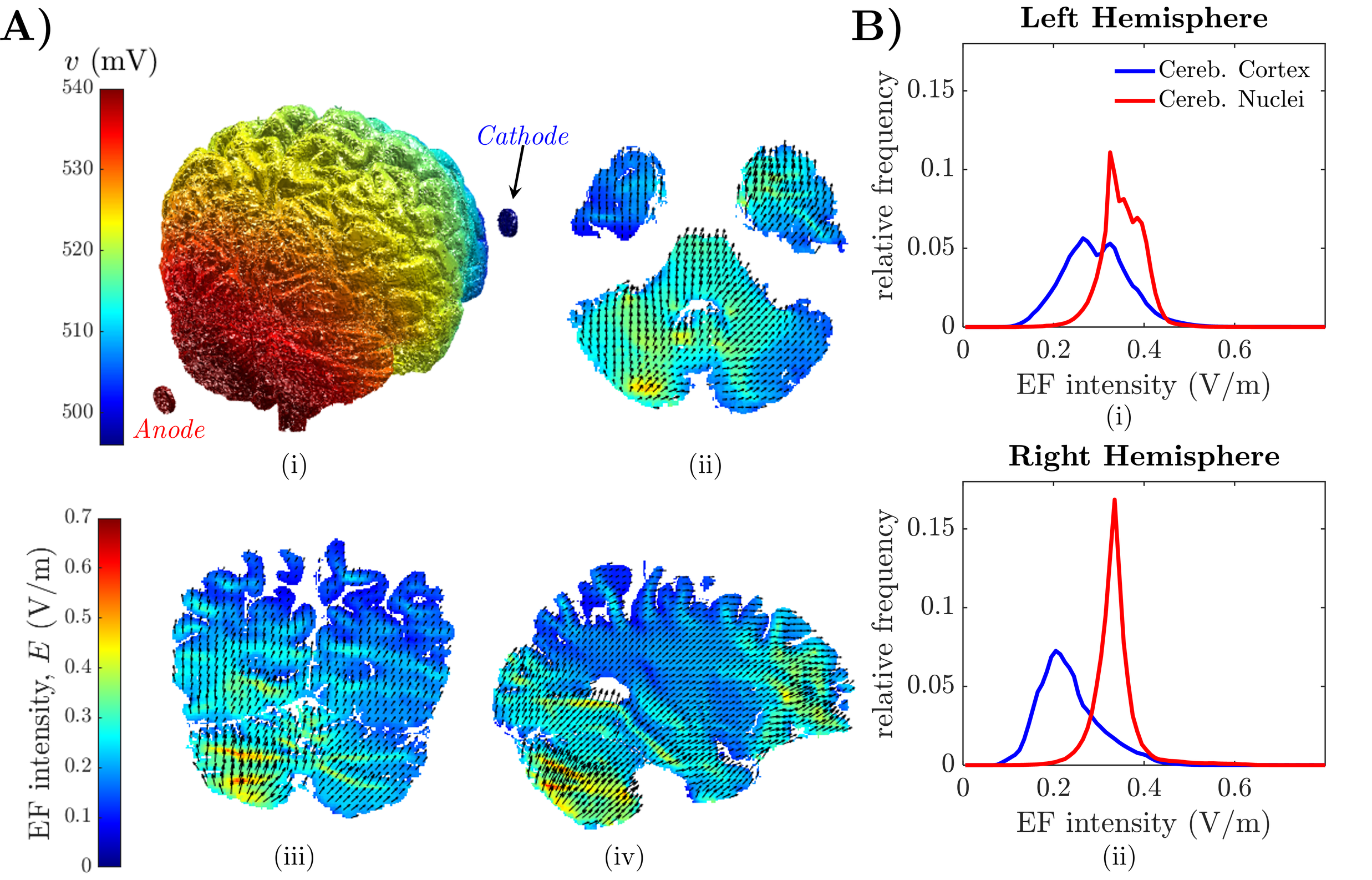

Supplement: S2 Fig — (A) Estimated intensity and orientation of the EF induced by 2-mA-cerebellar regular (R)-tDCS delivered with two ring electrodes in the brain for one human subject, i.e., atlas 5 from [26]. Panel (i) reports the position of the anode and cathode. Panels (ii), (iii), and (iv) report an axial, coronal, and sagittal view of the EF distribution. Colormap in (i) indicates the distribution of the electric potential, v (scale on the left). In (ii)-(iv), colormaps indicate the EF intensity, and black arrows indicate the EF orientation. Color scale in (iii) also applies to (ii) and (iv). (B) Sample distribution of the EF intensity for the R-tDCS montage in (A). Sample distributions are reported for the left (target) cerebellar hemisphere (i) and the right cerebellar hemisphere (ii), respectively. Distribution functions are computed separately for voxels mapping the cerebellar cortex (blue lines) and the cerebellar nuclei (red lines). EF intensity in the left hemisphere is higher compared to the right hemisphere, both for the cortex and the nuclei (one-way ANOVA test after Bonferroni correction, P-value P<0.001). Results in this figure can be directly compared with the results in Figs 1 and 2. Graphs in this figure were generated as reported for Figs 1 and 2. (TIF) [file pcbi.1009609.s002.tif]

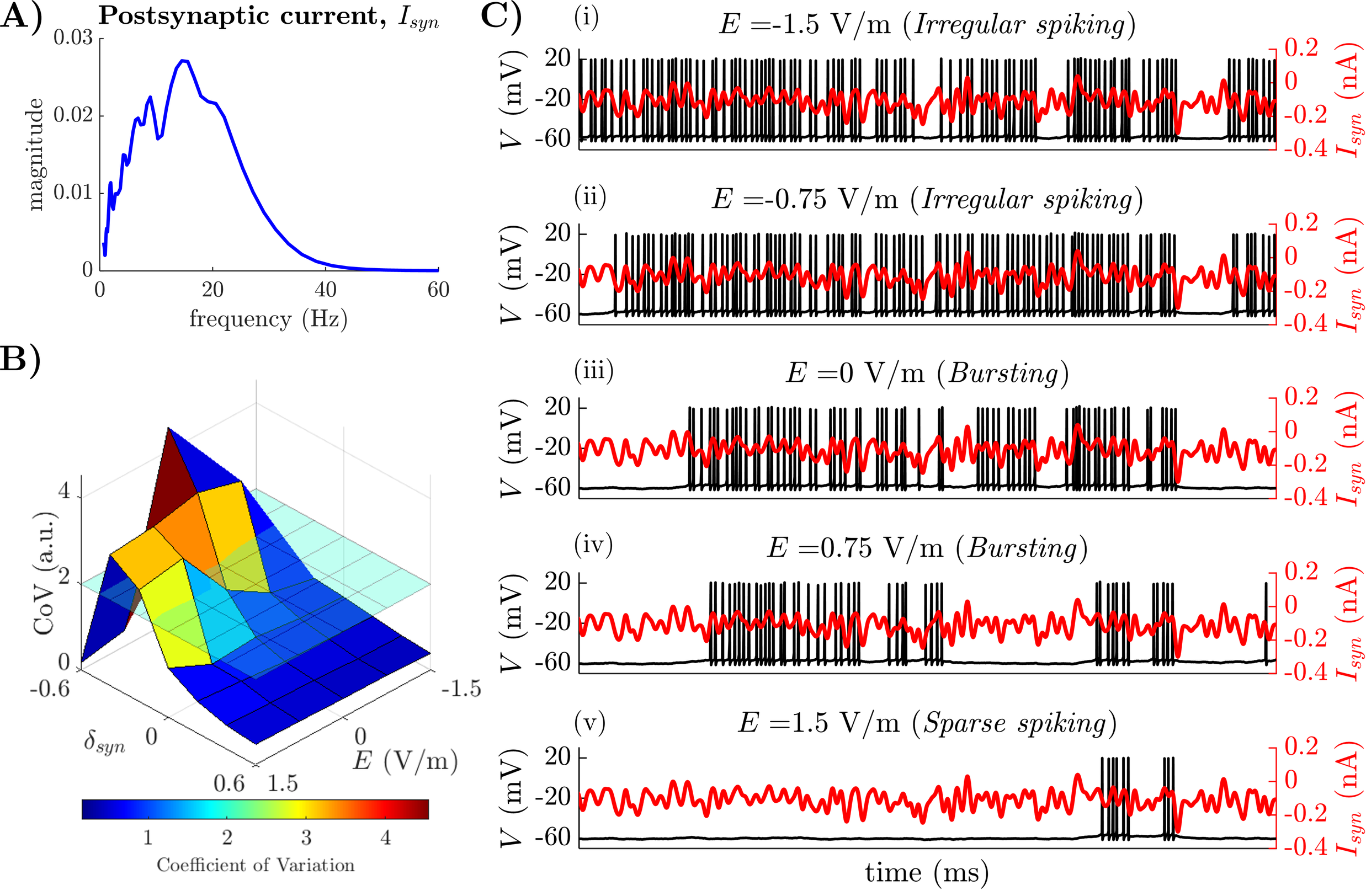

Supplement: S3 Fig — (A) Normalized scalogram of the weighted sum of 1,000 glutamatergic and 100 GABAergic synaptic activation sequences applied on the dendrites of the PC model to generate the results in Fig 5. The summed sequence is: S = gGABA(60+EGABA)hGABA−gglut(60+Eglut)hglut, where gglut, Eglut, and hglut are the synaptic conductance, reversal potential, and time histogram (bin size: 1 ms) of the activation sequence for the glutamatergic synapses, respectively; gGABA, EGABA, and hGABA are the synaptic conductance, reversal potential, and time histogram for the GABAergic synapses, respectively. The scalogram was computed via continuous wavelet transform (Morse kernel, sampling frequency: 1,000 Hz), filtering with a 30-Hz low-pass filter, and averaging samples over time. (B) Coefficient of variance (CoV) of the inter-spike intervals estimated at the PC soma for different combinations of values for parameter δsyn and R-tDCS-induced EF intensity, E. Pairs (δsyn, E) resulting in dense and irregular spiking as defined in the main text are characterized by low CoV (i.e., <2; below the opaque green plane) while pairs resulting in bursting and sparse spiking are characterized by high CoV values (>2; above the opaque green plane). CoV was not computed for δsyn = – 0.6 and E = 0.75 or δsyn = – 0.6 and E = 1.5V/m due to too few spikes in the entire spike train. (C) Transmembrane voltage at the PC soma (black lines) and estimated total synaptic current (red lines) under tDCS-induced EF at -1.5 (i), -0.75 (ii), 0 (iii), 0.75 (iv), and 1.5 V/m (v), respectively, and δsyn = – 0.4. Negative and positive EF intensities correspond to anodal and cathodal stimulation, respectively. Total synaptic current (right vertical axis) is estimated for -60-mV-membrane potential. Negative values indicate that the total synaptic current is hyperpolarizing. The results presented here complement the analysis reported in Fig 5. (TIF) [file pcbi.1009609.s003.tif]

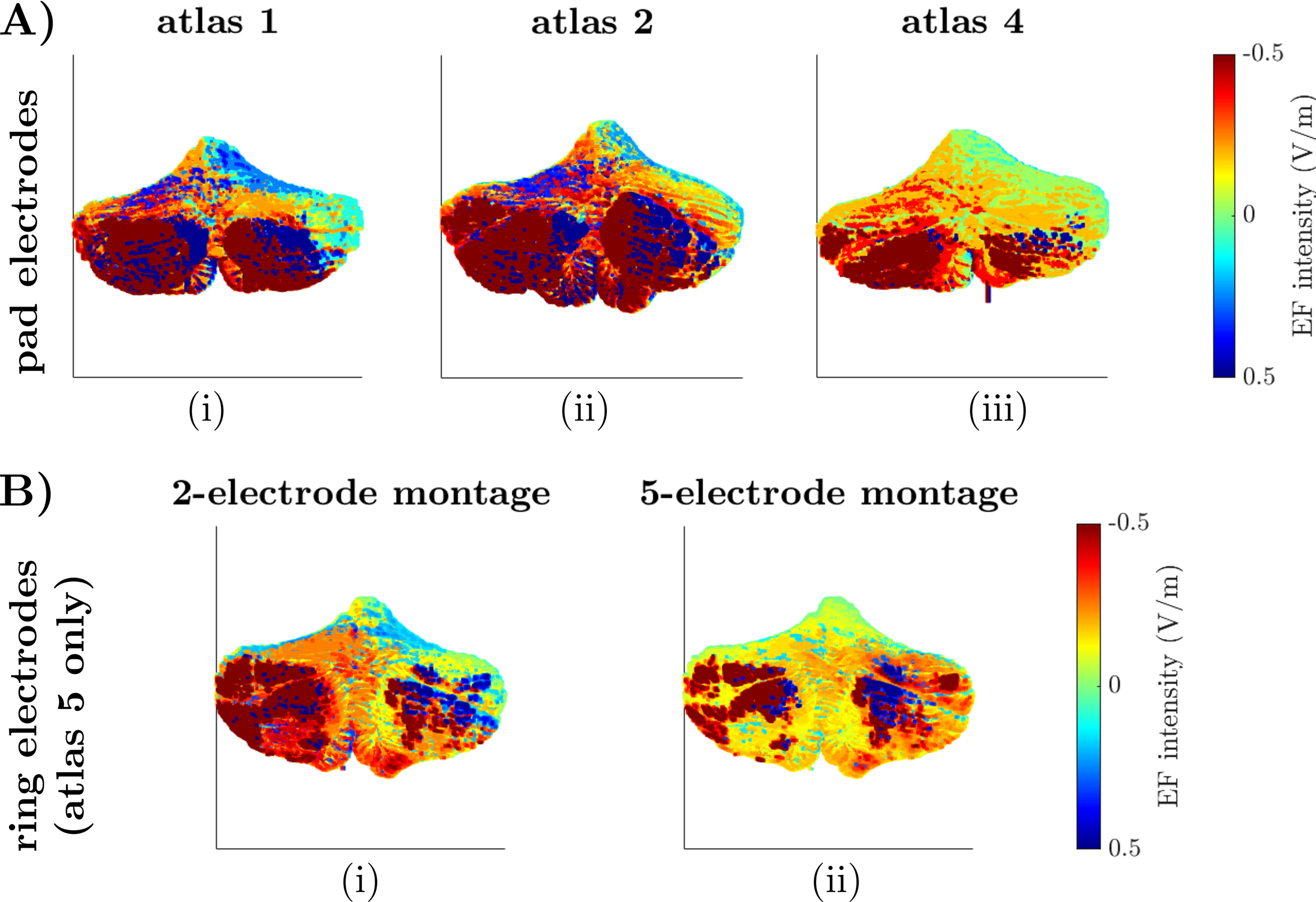

Supplement: S4 Fig — (A) Spatial distribution of the projections of the electric field (EF) onto the somato-dendritic axis of the Purkinje cells along the cerebellar cortical surface in three subjects, i.e., atlas 1 (i), atlas 2 (ii), and atlas 4 (iii) from [26], respectively. EF is generated for regular 2-mA-tDCS with pad electrodes, i.e., same configuration as in Fig 1A. (B) Spatial distribution of the EF projections onto the somato-dendritic axis of the Purkinje cells along the cerebellar cortical surface of one subject, i.e., atlas 5 from [26], in case of regular tDCS with two ring electrodes (i) and high-density tDCS with five electrodes (ii), respectively. Montages in (i) and (ii) are as in Figs S2A and 1B, respectively. Color scale on the right applies to all plots in (A-B). These plots complement the results reported in Fig 8. (TIF) [file pcbi.1009609.s004.tif]

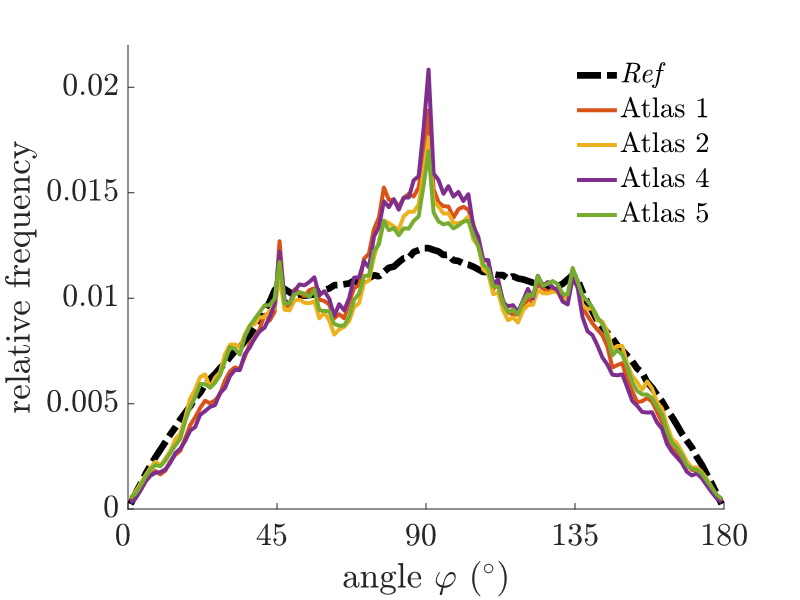

Supplement: S5 Fig — Sample distribution function of the angle φ between a preset vector pointing 45° between the anterior and superior axes towards the cerebellar midline and the normal vector on the cerebellar surface for four subjects (i.e., atlas 1, 2, 4, 5 from [26]) and the sample high-resolution atlas (Ref) presented in [119]. (TIF) [file pcbi.1009609.s005.tif]
