# Supplementary material for: Transcranial direct current stimulation of cerebellum alters spiking precision in cerebellar cortex: A modeling study of cellular responses
Source: PLoS Comput Biol. 2021 Dec 9;17(12):e1009609. doi: 10.1371/journal.pcbi.1009609 (PMC8691604; doi:10.1371/journal.pcbi.1009609)
Supplement: S1 Text — The text describes the procedures used to generate the graphs in S5 Fig and assess the effects of segmentation on the orientation of the cerebellar surfaces. (DOCX) [file pcbi.1009609.s010.docx]

**S1 Text**

***Transcranial Direct Current Stimulation of Cerebellum Alters Spiking Precision in Cerebellar Cortex: A Modeling Study of Cellular Responses***

Xu Zhang^1,2,3^, Roeland Hancock^2,3,4^, Sabato Santaniello^1,2,3*^

^1^ Biomedical Engineering Department, University of Connecticut, Storrs, Connecticut, United States of America, ^2^ Brain Imaging Research Center, University of Connecticut, Storrs, Connecticut, United States of America, ^3^ Connecticut Institute for the Brain and Cognitive Sciences, University of Connecticut, Storrs, Connecticut, United States of America, ^4^ Department of Psychological Sciences, University of Connecticut, Storrs, Connecticut, United States of America

* [sabato.santaniello@uconn.edu](mailto:sabato.santaniello@uconn.edu)

# **SUPPLEMENTARY METHODS**

To assess whether our results were biased by the MRI segmentation resolution, or the segmentation method used to isolate the cerebellar cortical surface, we compared the distributions of the angle $\varphi$ between a preset vector pointing 45° between the anterior and superior axes (i.e., towards the cerebellar midline) and the normal vector on the cerebellar surface. We measured $\varphi$ for all subjects in our study (i.e., atlases 1, 2, 4, and 5 from ref. [1]) and for the reference cerebellar surface (*Ref*) provided by Sereno *et al*. in [2]. The surface *Ref* was processed in FreeSurfer [3] to obtain the boundaries of the pial area surrounding the cerebellar region, and the surface normal vectors along the cerebellar cortices were computed in MATLAB using the function **patchnormals** [4],

**S5 Fig** shows that the distributions obtained in ref. [2] and in our study are highly overlapped and symmetric, with a peak at 90° and similar kurtosis values (*Ref*: 2.07; atlas 1, 2, 4, and 5: 2.25, 2.16, 2.30, and 2.15, respectively, 2.22 ± 0.07, mean ± S.D.). Also, angles $\varphi$ in the ranges [0°, 60°] and [120°, 180°] only account for a difference of 4.5% between the average distribution of the four atlases and the *Ref* distribution, whereas angles in the range [60°, 120°] amount to a difference of 7.38%. Given that the actual projected field values are minimal around 90° and contribute modestly to the stimulation effects, we concluded that the MRI image resolution and the cortical surface segmentation method had negligible effects on the calculation of the projected field values.

**SUPPLEMENTARY REFERENCES**

1. Park MT, Pipitone J, Baer LH, Winterburn JL, Shah Y, Chavez S, et al. Derivation of high-resolution MRI atlases of the human cerebellum at 3T and segmentation using multiple automatically generated templates. Neuroimage. 2014;95:217-31. Epub 2014/03/25. doi: 10.1016/j.neuroimage.2014.03.037. PubMed PMID: 24657354.

2. Sereno MI, Diedrichsen J, Tachrount M, Testa-Silva G, d'Arceuil H, De Zeeuw C. The human cerebellum has almost 80% of the surface area of the neocortex. Proc Natl Acad Sci U S A. 2020;117(32):19538-43. Epub 2020/07/30. doi: 10.1073/pnas.2002896117. PubMed PMID: 32723827; PubMed Central PMCID: PMCPMC7431020.

3. Fischl B. FreeSurfer. Neuroimage. 2012;62(2):774-81. Epub 2012/01/18. doi: 10.1016/j.neuroimage.2012.01.021. PubMed PMID: 22248573; PubMed Central PMCID: PMCPMC3685476.

4. Kroon D-J. Patch Normals Nantic, MA: Mathworks, Inc.; 2009 [cited 2020 October 27,]. 1.0.0.0:[Calculates the normal vectors of a triangulated mesh (patch)]. Available from: <https://www.mathworks.com/matlabcentral/fileexchange/24330-patch-normals>.
