# Supplementary material for: Transcranial direct current stimulation of cerebellum alters spiking precision in cerebellar cortex: A modeling study of cellular responses
Source: PLoS Comput Biol. 2021 Dec 9;17(12):e1009609. doi: 10.1371/journal.pcbi.1009609 (PMC8691604; doi:10.1371/journal.pcbi.1009609)
Supplement: Attachment — Submitted filename: R2R_Review_PCOMPBIOL-D-20-02326.pdf [file pcbi.1009609.s011.pdf]

Editor's Remarks to Authors:

We are fortunate to have received reviews of three experts and in light of the reviews, we would like to invite the resubmission of a significantly revised version that takes into account the reviewers' comments.

We cannot make any decision about publication until we have seen the revised manuscript and your response to the reviewers' comments. Your revised manuscript is also likely to be sent to reviewers for further evaluation.

Authors: We sincerely thank the editors and reviewers for their work on this manuscript. We have carefully revised the study and expanded the scope of our work to address the excellent points raised by the reviewers. Accordingly, the manuscript has been carefully edited to reflect the additional work and better support the conclusions of our study. We report a complete list of replies to the reviewers' comments in the following subsection.

Reviewer Comments:

Reviewer #1:

In the paper of Zhang *et al*., the authors investigate the effects of transcranial direct current stimulation in cerebellum using MRI and modelling of electric fields and multi-compartment models of neurons. It proposes cerebellar tDCS as a therapeutic option for disorders involving cerebellar hyperactivity such as ataxia.

Overall, the paper is well written. The main objectives and findings are clearly laid out. The findings are interesting and are well explained to the reader. Below are my specific comments.

Authors: We sincerely thank the reviewer for the feedback. Our replies to the reviewer's comments and concerns are reported below.

Major Comments:

1) Three multicompartment neurons models have been used in this study and one of them has a simple morphology (GrC). Why does GrC have a simple morphology compared to the other ones? I would suggest having additional realistic morphologies, or to have three full complex morphologies at least.

Authors: We agree with the reviewer that using a simplified morphology can negatively impact the conclusions of the study. To address this issue, we replaced the original, simplistic granule cell (GrC) model, which was first proposed in (Diwakar et al., 2009), with a new model. The revised model has a full complex morphology, which was reconstructed from a human GrC. The new model is summarized in Fig. 2B (*ii*) and the subsection "*Cerebellar neuron models*" in the revised manuscript (line 748-788).

Briefly, the new model is multi-compartment and incorporates the detailed 3D geometry of the soma and dendritic tree of a granule cell from the NeuroMorpho.org database (ID: NMO_32569). The cell morphology is characterized by larger somatic surface and longer somato-dendritic length

than the morphology of the model by (Diwakar et al., 2009), i.e., somatic surface: 217.27μm^2 vs. 105.68μm^2; somato-dendritic length: 83.51μm vs. 20.80μm; new model vs. old model. In addition, the model is completed with an ascending axon and hillock, where the axon is orthogonal to the dendritic tree. The models for the ascending axon and hillock are in (Diwakar et al., 2009). Finally, glutamatergic synapses were placed on the dendritic tree as proposed in (Masoli et al., 2020) to mimic the activation patterns evoked by the presynaptic activity of mossy fibers.

Altogether, soma and dendrites in the new cell model occupy a combined volume of 589.31μm^3 and were discretized into 26 compartments (i.e., 25 dendrites and one soma) by using CellBuilder in NEURON (Carnevale and Hines, 2006). The equivalent diameter and length of each compartment were determined in CellBuilder automatically, based on the cartesian coordinates and diameter of the cell sections reported in dataset NMO_32569. On average, the compartment length was 19.8 ± 18.9μm (mean ± S.D., *n*=26 compartments), which is comparable to the mean compartment length of the other two models included in our study, i.e., Purkinje cell and deep cerebellar neuron.

Somatic, dendritic, and peri-axonal compartments of the new GrC model were endowed with membrane capacitance, ionic currents, leakage current, and cytoplasmic resistivity. Ionic channel kinetics were modeled as in (Diwakar et al., 2009) and the values of cytoplasmic resistivity, membrane capacitance, and ionic channel conductance were adapted from the values in (Diwakar et al., 2009) to preserve the matching between numerical simulations and *in vitro* patch-clamp recordings of granule cells. Specifically, parameters in our model were adjusted to match the current-frequency curve reported in (Diwakar et al., 2009), which was fit on *in vitro* recordings of granule cells. The values of parameters in our model are reported in Table S3 in the *Supplementary Information* document submitted along with the revised manuscript.

The new GrC model was used to further expand our investigation of the effect of tDCS. Specifically, we added presynaptic stimuli to one dendritic branch as proposed in (Masoli et al., 2020) to replicate the adaptive spiking pattern that granule cells exhibit in response to mossy fibers. Then, we quantified the changes to the spiking pattern at the soma and the axon that occur under cathodal and anodal EF. Results of these additional simulations are reported in Fig. 4B-C and subsection "*GrC responses to mossy fiber activation are robust against tDCS*" in the revised manuscript (line 268-304).

Overall, the additional simulations confirm that, despite an enlarged geometry and time-varying synaptic stimuli, the dynamics of the granule cells are only modestly affected by applied electric fields up to 1.5 V/m, likely because of the ion channel composition and location of these neurons, which are placed in a deeper layer underneath the molecular layer and Purkinje cell layer (Consalez et al., 2020).

2) Moreover, we are not given many details about these morphologies (especially for the intrinsic dynamics).

Authors: The subsection "*Cerebellar neuron models*" and two supplementary tables in the *Supplementary Information* document (i.e., Table S2 and Table S3) have been added to provide details about the neural morphologies and intrinsic ionic channel dynamics. Please, see line 748-788 in the revised manuscript.

3) Some figures (Figs. 1 and 2) are quite difficult to read clearly (too many subpanels) and/or we

are given too much information not necessarily pertinent. I would suggest keeping key information/graph. For example, no need of the 3 polarization patterns on Figure 1.C (i-iii). Just one would be enough to illustrate the neuron polarization.

Authors: We thank the review for the suggestion. Figures have been carefully revised in the manuscript to increase fonts, reduce the number of subpanels, and better emphasize the key information in each graph. Graphs that report minor results or nonessential details are now available in the *Supplementary Information* document submitted along with the revised manuscript.

Minor Comments:

- *Line 85*: Add the reference radman2009cortical.

Authors: Thank you. The reference (Radman et al., 2009) has been added.

- *Line 150*: Finding higher electric field intensity in the cerebellar nuclei than in in the cerebellar cortex was expected?

Authors: We expected different electric field intensities in the cerebellar nuclei compared to the cerebellar cortex. Our expectation is motivated by the difference between the tissue conductivity values of the cerebellar white matter and the cerebellar gray matter.

Specifically, we programmed the ROAST finite element modeling solver as recommended by the authors in (Huang et al., 2019) and assigned a conductivity value of 0.126 S/m to white matter tissues, including the cerebellar cortices, and a value of 0.276 S/m to gray matter tissues, including the cerebellar nuclei. The conductivity values used in our simulations are as in (Huang et al., 2013) and are now reported in the subsection "*Cerebellar tDCS induces mild electric fields across cerebellum*" of the revised manuscript, line 132. Moreover, Table S1 in the *Supplementary Information* document now reports the conductivity values assigned to all tissues and layers used in our ROAST simulations.

- *Line174*: How much lower? It is significant but giving some numbers would be appreciated.

Authors: The variation in average field intensity was approximately 49% as we transitioned from regular tDCS to HD-tDCS, i.e., the average field intensity was 0.40 ± 0.27 V/m across the cerebellar tissues for the regular tDCS and 0.20 ± 0.15 V/m for HD-tDCS (mean ± S.D.).

We now report both the percentual variation and the average field intensity values for regular tDCS and HD-tDCS on line 165-172 in the revised manuscript. In addition, the probability distribution functions of the electric field (EF) intensity values evoked by regular tDCS and HD-tDCS can be found in Fig. 2A and Fig. 2B, respectively, in the revised manuscript.

- *Line 185*: Previous studies have introduced the polarization length. Explain it in short sentence and add references.

Authors: We followed the definition provided in (Radman et al., 2009) and defined "*polarization length*" of a cell as the cell-specific subthreshold somatic polarization per unit electric field applied. Since the subthreshold polarization is measured in mV and the electric field in mV/mm,

the polarization length is expressed in millimeters. This definition is now reported in the revised manuscript, on line 222.

- *Line 199*: The firing rate of some cells was not highly affected by the tDCS was also demonstrated by previous studies.

Authors: The reviewer is correct. (Asan et al., 2020) recently showed *in vivo* that cerebellar DC fields less than 1.5 V/m cause a modest modulation of the average firing rate of Purkinje cells, with the modulation mainly consisting of strong transients at the stimulation onset and offset and an intervening plateau. These results confirmed earlier findings by Chan and Nicholson, who investigated the response of both Purkinje cells and stellate cells to DC fields *in vitro* (Chan and Nicholson, 1986). We now acknowledge this study on line 229 in the revised manuscript.

- *Line 205*: Give some reasons to explain the difference of polarization of cells (morphologies…)

Authors: Large cells like Purkinje cells have lower axial resistance compared to small cells like granule cells due to larger compartment diameters. This is reflected in our multicompartment models, e.g., see Table S2 in the *Supplementary Information* vs. Table 1 in (Masoli et al., 2015). Accordingly, the length constants for Purkinje cells are longer compared to granule cells, and this determines higher polarization in Purkinje cells than granule cells (Svirskis et al., 1997). This is now explained on line 231-234 in the revised manuscript.

- *Line 246*: The firing rate value. Is there a specific reason to have a firing rate of ~100Hz?

Authors: We report the *instantaneous* firing rate of the deep cerebellar neuron (DCN), which is calculated as in (Steuber et al., 2011) by interpolating the reciprocal values of the DCN's interspike intervals. In our simulations, a high instantaneous rate, i.e., 100 Hz or higher, is consistent with values reported in (Steuber et al., 2011) and occurs at the beginning of the after-hyperpolarization rebound firing. High instantaneous rates, in fact, capture the adaptation in firing rate that DCNs exhibit during after-hyperpolarization bursting.

- *Line 264*: Linear relation. What is the correlation value? Coefficient of determination R^2?

Authors: The correlation value R is 0.985. The coefficient of determination R^2 is 0.97. These values are now reported in the revised manuscript, on line 313-314.

- *Line 295*: How is the threshold of 80 ms chosen? Same question for the 80-200ms range. Add reference for these values.

Authors: The firing patterns of Purkinje cells were primarily classified according to the coefficient of variation (CoV) of the inter-spike interval (ISI) sample distribution, i.e., a pattern was defined as "*dense*", "*irregular*", or "*bursting*" depending on whether CoV≤ 0.5, 0.5< CoV≤ 2, and CoV >2, respectively. The thresholds on CoV defining the transition between firing modes were adjusted from the values proposed in (Robin et al., 2009).

Following the classification based on CoV, we observed that spiking patterns with CoV≤ 0.5

(i.e., dense spiking) were characterized by ISI< 80ms and spiking patterns with 0.5< CoV≤ 2 (irregular spiking) had ISI values up to 200ms. Finally, we observed that, among the spiking patterns with CoV>2, tonic bursting was associated with inter-burst intervals around 200ms while ISI values of exceptional length (i.e., above 3,000ms) would mainly occur in case of sporadic firing. Hence, we introduced an additional criterion (i.e., CoV>3 and maximum ISI value greater than 3,000ms) to identify "*sparse*" spiking. The latter criterion was set heuristically based on the inspection of cases with CoV >2, i.e., cases indicated by green and gray dots in Fig. 5E in the revised manuscript.

Note that we used the coefficient of variation instead of a surprise index like in (Legendy and Salcman, 1985) to define burstiness because of the correlation that has been established between CoV and surprise indices in bursting mode, see (Robin et al., 2009). We used, instead, a heuristic rule to define sparse spiking because Purkinje cells rarely fire a single, isolated action potential, e.g., see experiments in (Jaeger and Bower, 1994; Khaliq et al., 2003). As reported in Fig. 5C (iv) in the manuscript, in fact, sparse spiking neurons were characterized by a handful of brief volleys of action potentials, with a prolonged silence between consecutive volleys.

We have now included relevant references to explain the classification of firing patterns. Please, see line 348-357 in the revised manuscript.

- *Line 307*: Units of the slope.

Authors: The units of the slope are now reported.

- *Line 314*: '*overall limited… within ± 25%*'. This number is quite a lot for a firing rate variation so talking about 'limitation' is not entirely correct.

Authors: We agree with the reviewer. The statement has been modified. Please, see line 365-368 in the revised manuscript.

- *Line 327*: Has this soma transition been observed in previous studies (modeling or *in vitro*)?

Authors: The somatic transition from an irregular pattern to a more regular, entrained pattern under the effects of applied electric fields has been observed *in vitro*, e.g., (Chan and Nicholson, 1986; Asan et al., 2020). Interestingly, alternate electric fields were used in these studies instead of constant fields. Hence, our simulations indicate an alternative way to shift the firing pattern of Purkinje cells, i.e., through constant electric fields. Differently from alternate fields, it is unlikely that constant fields would generate entrainment among Purkinje cells, but they could steer the cells to discharge patterns with similar statistical properties over a large region of cerebellar cortex.

These considerations and additional references have been included in the revised manuscript, on line 405-413.

- *Line 368*: Is there a general method to characterize complex spikes? (Threshold, number of spikelets, time latency, …).

Authors: Yes, complex spikes are generally characterized according to (Lang et al., 2014) as

bursts of spikes fired at high frequency, in which the initial spike is followed by small spikelets with varying numbers and amplitudes. We now refer to (Lang et al., 2014) in our manuscript for a definition of "*complex spike*".

- *Line 396*: Why specifically focusing on the Na^+ v1.6 dynamic? Did previous study investigate a relation between the silent period and this channel? Or more generally, the ionic channels? If yes, add reference.

Authors: *In vitro* experiments reported in (Khaliq et al., 2003) showed that Na^+ v1.6 channels in Purkinje cells are responsible for the initiation of simple spikes and mediate the rapid recovery from inactivation, thus controlling the duration of the silent period. Hence, we specifically focused on the Na^+ v1.6 dynamics.

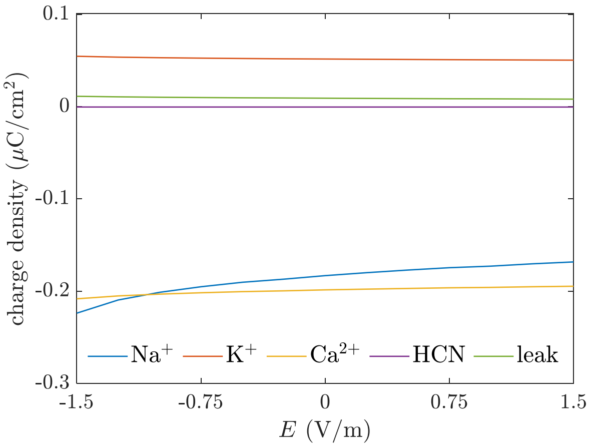

Figure R1. Charge density of different ionic currents measured at the soma of the PC model during silent period (T_{sp}) under constant electric fields versus the field intensity (E). Ionic currents include leakage currents (leak) and currents flowing through sodium (Na^+) Nav1.6 channels, hyperpolarization activated cyclic nucleotide-gated cationic (HCN) channels, potassium (K^+) channels (combination of Kv1.1, Kv1.5, Kv3.3, Kv3.4, Kv4.3, KCa1.1, KCa 2.2, KCa 3.1, and Kir2 channels), and calcium (Ca^{2+}) channels (i.e., combination of Cav3.1, Cav 3.2, Cav 3.3, and Cav2.1 channels). See (Masoli et al., 2015) for a definition of the ionic channel mechanisms. Since the duration of T_{sp} changes with the field intensity E, we computed the charge density as the integral of the ionic current density from the end of the complex spike to the end of the shortest T_{sp}, i.e., 251 ms.

Moreover, we looked at the dynamics of all somatic channels during a silent period, both at rest and under electric fields (EF), and we observed that Na^+ v1.6 channels were the most sensitive to the EF intensity. This is reported in **Figure R1** here, which shows the charge density carried by different somatic currents during the silent period under EF up to 1.5 V/m. **Figure R1** reports that, while the charge density of the potassium and calcium currents is modestly affected by EF, sodium currents through Nav1.6 channels reported a remarkable excursion as the polarity of the EF varied. This is further quantified by measuring the absolute difference, Δc, between the charge density of each ionic current measured under -1.5 V/m EF (anodal field) and +1.5 V/m EF (cathodal field). The values of Δc for Na^+, K^+, Ca^{2+}, cationic (HCN), and leakage (leak) currents are reported in **Table R1**. These values show that the variation in Na^+ currents under EF is more than 4 times larger than the remaining ionic currents. This information and the reference (Khaliq et al., 2003) are now reported in the revised manuscript at line 441-445 and Table S4 in the *Supplementary Information*.

**Ionic Channels**	**Δc (μC/cm^2)**
Na^+	5.55×10^{-2}
K^+	4.20×10^{-3}
Ca^+	1.36×10^{-2}
HCN	3.08×10^{-5}
leak	3.10×10^{-3}

Table R1. Absolute difference between the charge density in Figure R1 estimated for E =-1.5 V/m and E =+1.5 V/m. Difference is computed for sodium (Na^+) channels, hyperpolarization activated cyclic nucleotide-gated cationic (HCN) channels, potassium (K^+) channels, calcium (Ca^{2+}) channels, and leakage (leak) channels.

- *Line 453*: Results with the dendritic polarization. The authors claim that '*the dendritic polarization* [...] *modestly changed the burst count and the mean period*'; on the Figs. 4.E and 4.F, the green line shows almost no difference with the amplitude of E – especially for the burst count. We could see a light decrease of the T_{burst} for amplitude above 0.6 though.

Authors: We thank the reviewer for noticing this. We have edited the text accordingly. Please, see line 486-489 in the revised manuscript.

- *Line 518*: Distribution between 30 and 150 degrees. Give a percentage of the distribution (70%, 80% are in this range?)

Authors: Approximately 86.8% of the distribution fell between 30 and 150 degrees. This value is now reported on line 537 in the revised manuscript.

- *Line 520*: Concerning the Supplementary Fig. S5, why can we observe two symmetric spikes around 45 and 135 degrees?

Authors: The peaks observed at 45 and 135 degrees are likely determined by the voxel resolution used in our pipeline, i.e., 600µm. When applied to the MRI atlases from (Park et al., 2014), in fact, this voxel resolution often resulted in steep edges along the contour of the reconstructed cerebellar folds, which increased the likelihood of having angles between the EF and the Purkinje cell somato-axonal axis close to 45°, 90°, or 135°.

- *Line 578*: Pyramidal cells are known for being quite responsive to electric field (tACS or tDCS) and more responsive than inhibitory cells or interneurons. Even for low amplitude (~1mV/mm)

Authors: We agree with the reviewer and have therefore revised the statement. Please, refer to line 588-591 in the revised manuscript.

- *Line 609*: The firing pattern not only depends on the intensity of electric field but also on the morphology of the cell or the ionic channels. It would be interested to investigate these parameters in Purkinje cells. Any idea on the influence of these parameters?

Authors: We agree with the reviewer that the cell morphology and ionic channels could affect the firing pattern of Purkinje cells. Since this important topic falls outside the scope of the current study, we have added a paragraph in the DISCUSSION section where we review the potential impacts that the cell morphology might have on our results. Please, see line 630-636 in the revised manuscript.

Briefly, recent *in vivo*, *in vitro*, and computational studies have converged in showing that the spiking pattern of the Purkinje cells is critically shaped by the synapses formed by climbing fibers and interneurons on the cells' dendritic spines (Solinas et al., 2006; Pan et al., 2020). Hence, it is plausible that the morphology of the dendrites can influence the firing pattern of the Purkinje cells by modulating the efficacy of the synaptic transmission. This is further supported by (Harris and Stevens, 1988), which showed *in vitro* that the efficacy of synaptic transmission depends on the size of the dendritic spine heads. Furthermore, (An et al., 2019) compared the discharge pattern of various Purkinje cell morphologies obtained from different animal models. In this study, authors reported that the dendritic morphology of the Purkinje cells can affect both the shape of the somatic action potentials and the accuracy of the response to synaptic stimuli from parallel fibers.

While this evidence suggests that the cell morphology can alter the Purkinje cell's discharge

pattern, we should note that, in (An et al., 2019), the cell morphology affected the mean firing rate of the Purkinje cells, but no significant alteration of the shape and variance of the sample distribution of the inter-spike intervals was reported. Also, (Harris and Stevens, 1988) reported that the spine length and volume vary modestly across the dendritic tree of Purkinje cells, with this trend being consistent across animal species.

Altogether, this evidence suggests that, under applied EF, modifications to the Purkinje cell's morphology could alter the discharge patterns compared to those patterns proposed in our study, but these alterations would likely be limited to the average firing rate. Hence, we expect that morphology-related changes to the Purkinje cell's firing activity will likely result in discharge patterns that still fall within the classification scheme shown in Fig. 5. Perhaps more importantly, we expect that the modified discharge patterns would still transition from one class to one another according to the scheme reported in Fig. 5E in our study.

- *Line 652*: Add a paragraph about the clinical application of the findings and how it can be related to brain disorders.

Authors: The subsection "*Translational implications for clinical applications*" has been added to the DISCUSSION to better elaborate on the potential clinical applications of our results. In this subsection, please consider the additional paragraph that is reported on line 700-716 in the revised manuscript.

- *Line 700*: 3D multi-compartment models have been used but we do not know many details about them. Give more details about the dynamics (active soma (Hodgkin Huxley?), passive dendrite.…), ionic channels modeling.

Authors: We have added a section titled "*Cerebellar neuron models*" in the revised manuscript (see line 748-788). This subsection now provides a brief introduction to the neuron models and the biophysical properties of their compartments. The original articles, where these models were first presented, are also cited in this section to complement the presentation.

- *Line 704*: Cerebellar interneurons and Golgi cells have been omitted for some reasons, but it would be interesting to see how they are affected by the electric field (in supplementary with preliminary results or in the next paper).

Authors: We agree with the reviewer that the effects of cerebellar tDCS on interneurons and Golgi cells could be important, especially because interneurons are expected to regulate the inhibition of Purkinje cells.

We excluded Golgi cells and cerebellar interneurons from the study for two reasons. First, the focus of our work was limited to those cerebellar neurons that have large size and/or present a wide dendritic arborization. The models considered in our study, i.e., deep cerebellar neurons, Purkinje cells, and granule cells, in fact, are all characterized by relatively large somas, long axons, and highly developed dendritic trees. Also, Purkinje cells and granule cells are in the outer layers of the cerebellum, while deep cerebellar neurons receive direct projections from the Purkinje cells. Altogether, we expected that these three cell types would be the primary target of cerebellar tDCS, i.e., the neurons that would exhibit the largest response to the transcranial stimulation.

On the other hand, Golgi cells and cerebellar interneurons are among the smallest neurons in the cerebellum, have limited dendritic span, and are scarcely arranged within the molecular layer of the cerebellum (Leto et al., 2006). Hence, we expected that the effects of cerebellar tDCS on these neurons would be subtle and lesser noticeable than the effects on Purkinje cells, granule cells, and deep cerebellar neurons.

The second reason that advised against including Golgi cells and cerebellar interneurons in this study is that we primarily focused on the effects of tDCS on neural excitability, i.e., on how neurons would respond to presynaptic stimuli. In this scenario, we had to keep the presynaptic stimuli independent of the applied electric field, and therefore we assumed that the effects of tDCS on the interneurons, which project onto Purkinje cells, were negligible. However, we expect to continue this line of work in a follow-up study, which will specifically focus on the effect of tDCS-induced EF on mossy fibers, climbing fibers, and parallel fibers.

A brief explanation of the rationale behind our selection of neurons is now reported in the section "*Cerebellar neuron models*" of the revised manuscript. Please, see line 750-756.

- *Line 712*: Why was the range ±1.5 mV/mm chosen? Is there any specific reason? Because the stronger electric field is, the stronger effects we will observe.

Authors: We set the maximum EF intensity at 1.5 V/m because of the predictions given by our computation in ROAST and analogous results reported in (Parazzini et al., 2014). In the latter study, authors estimated the distribution of EF intensity across the cerebellum and the occipital cortex when cerebellar tDCS is applied. They reported that, across three, patient-specific brains, the EF intensity in the cerebellar region was at or below 1.5 V/m. While other studies have estimated the EF in the cerebellum during cerebellar tDCS using different pipelines and approaches, e.g., see (Rezaee and Dutta, 2019), there has been a consensus across different approaches that tDCS-induced fields in the cerebellum should reach intensities below 1.5 V/m.

We have edited the manuscript to better explain the motivations for the range of field intensities. Please, see line 194-195 in the revised manuscript.

- *Line 731*: A GABAergic synapse is added to every dendritic compartment of the DCN model. How many are there?

Authors: A total of 400 GABAergic synapses were distributed on the dendritic compartments of the DCN model. In addition, 50 GABAergic synapses were placed on the soma, as proposed in (Steuber et al., 2011). This information is now reported in the revised manuscript on line 790-791.

- *Line 739*: E_{syn}=-90. How were this value chosen? Add reference.

Authors: The reverse potential, E_{syn}, of the GABAergic synapses applied on the deep cerebellar neuron model was chosen as in (Steuber et al., 2011). This study was the first to propose the model of deep cerebellar neuron that we use in our study. This information is now reported in the revised manuscript on line 797-798.

- *Line 808*: Add reference for these values.

Authors: Thank you for noticing this. The maximum conductance value for the glutamatergic synapses, g_{glut}, on Purkinje cells was kept at the baseline value of 25nS, which is the mid-point of the range of synaptic conductance values, 0.2-50.0nS, that has been reported in (De Schutter and Bower, 1994). The maximum conductance value for the GABAergic synapses, g_{GABA}, on Purkinje cells, instead, was kept at the baseline value of 100nS, which was chosen to reproduce the excitation/inhibition ratio of 0.25 as shown *in vitro* in (Mittmann and Hausser, 2007). This information is now reported in the revised manuscript on line 865-866.

Minor Comments on Figures:

- *All figures*: The font of the caption for each figure should be the same font as in the main body. Increase the font size too and the space.

Authors: Font size has been increased in the figures, and space has been added between consecutive subpanels. Thank you.

- *Fig 1.B*: So, there are 3 different types of cells (PC, GrC, DCN). What does the '*~700 μm*' or '*~20 μm*' refer to? The GrC does not have a realistic morphology (just a ball-and-stick and 4 dendrites) compared to the PC and DCN. Why?

Authors: The values '*~700 μm*' and '*~20 μm*' refer to the length of the axon and the diameter of the soma, respectively, of the Purkinje cell model.

Please, note that the images depicting the multicompartment models of Purkinje cell, deep cerebellar neuron, and granule cell, respectively, have been updated in the revised manuscript to better display the correct morphologies. In addition, a minimal set of geometric data, including the length of the axon and the diameter of the soma, has been added for the GrC model in Table S2 in the *Supplementary Information* document. Finally, please note that the granule cell model has been replaced in our study by a new model, which involves a realistic morphology, as described in the authors' reply to point 1) of this rebuttal.

- *Fig 1.B (i)*: What is 'NOR1, NOR2 and NOR3'?

Authors: The notation 'NOR #' indicates the node of Ranvier #, with #=1, 2, or 3, along the axon of the Purkinje cell model. The notation 'NOR #' is now removed from the revised image depicting the model of Purkinje cell used in our study. The total count of nodes of Ranvier in our model and the position of the nodes along the axon were chosen as in (Masoli et al., 2015).

- *Fig 1.C*: Add an arrow to show the direction of the electric field.

Authors: An arrow has been added in Fig. 2A to indicate the direction of the electric field along the neuron's somato-axonal axis. Thank you.

- *Fig 2.C*: What is the distance of the distal dendrite of the soma?

Authors: The linear distance between the most distal dendrite and the soma of the Purkinje cell model is 305.47µm. The value is reported on line 830 of the revised manuscript.

- *Fig 2.D*: For φ ~ 0.6, we have almost a perfect superposition of the 3 curves. How can you explain that? And why after that point, the red curve is under the black one and the blue line above the black one?

Authors: The phase response curves (PRC) estimated under anodal EF (-1.5 V/m) and cathodal EF (+1.5 V/m) have slightly different shapes, with the cathodal PRC predicting lower phase anticipation for stimuli applied at phases φ≤ 0.6 and slightly higher anticipation for stimuli applied at phases φ >0.6 compared to anodal tDCS. This flip in the relative position of the anodal PRC compared to the cathodal PRC is expected and likely due to the different firing rates of the Purkinje cell model under anodal tDCS and cathodal tDCS, respectively. As reported in (Couto et al., 2015), in fact, the shape and position of the PRC of a Purkinje cell are highly sensitive to the cell's inter-spike intervals, which are used as a proxy for the oscillation periods to estimate the PRC. More importantly, (Couto et al., 2015) showed that the phase anticipation predicted by the PRC at phases below 0.6 decreases as the firing rate increases. Vice versa, the phase anticipation predicted at phases above 0.6 increases with the firing rate, thus explaining the flip in relative position of the PRCs in our figure.

Furthermore, as our simulations transitioned from anodal tDCS to cathodal tDCS, the PC firing rate increased up to 3 Hz, which corresponds to an average reduction of approximately 2ms for the PC oscillation period. Such modest change in oscillation period can explain the similarity between the anodal PRC and cathodal PRC at phases above 0.6.

- *Fig 2.E (ii)*: What is the method to distinguish dense and irregular spiking?

Authors: Please, refer to our reply to the reviewer's comment regarding *line 295*. We have edited the example patterns reported in the subpanels of Fig. 5C. For each example, the value of coefficient of variation is now reported in the figure.

- *Fig 2.E (i)*: What is the ratio between excitatory/inhibitory synapses? Add it in the main text. Any reference?

Authors: Thank you for rising this point. The ratio between excitatory synapses and inhibitory synapses was set to 10:1 and reflects the fact that the number of excitatory synapses is severalfold larger than the number of inhibitory synapses on Purkinje cells, e.g., see (De Schutter and Bower, 1994). The specific value of the ratio was adjusted to reproduce the net excitation/inhibition ratio of Purkinje cells as reported in (Mittmann and Hausser, 2007). This information is now reported in the revised manuscript, on line 855-856.

Reviewer #2:

- Combined MRI-derived reconstruction of the cerebellum with a tDCS-induced EF.

- Multicompartmental cerebellar neurons: Purkinje cell, deep cerebellar neuron, granule cell.
- tDCS significantly modulates the post-synaptic spiking precision of PCs: Firing pattern, timing.
- No significant influence on DCN and GRC.
- tDCS modulated the synaptic inhibition, observed with discharge pattern, average firing rate, spiking pattern.
- The acute effects of tDCS on the cerebellum mainly focus on Purkinje cells and modulate the precision of the PC response to synaptic stimuli, thus having the largest impact when the cerebellar cortex is active.
- Method: FEM, HD-tDCS & pad-tDCS, 2mA, ROAST.

Strong Point: Considering the fact that most previous studies reported cortical neurons' response to tDCS, this research may be novel.

Weak Point: Overall, there is a lack of explanation or interpretation of results, i.e., give a reason and explanation with a citation. The implication of the result only is described. In addition, methodology was quite poorly explained (for example, two pad stimulation was applied, however, detailed head model used here, and configuration of electrodes were not presented. Authors addressed that ROAST was used only).

This study calculated a realistic EF range and applied the realistic EF to a multi-compartmental neuron model with a quasi-uniform assumption. This means there was a lack of information on the spatial distribution of neurons in a cerebellum, although the angle between EF and dendrite-soma-axon axis was considered. Comparing to multi-scale modeling, this approach is not realistic or advantageous.

Authors: We thank the reviewer for the assessment. We have carefully revised the manuscript, added critical details about the methodology, and included new text to better interpret the proposed results.

We understand the reviewer's concern about the spatial distribution of neurons and the potential advantages of our approach, and we would like to further clarify our work.

Firstly, the spatial distribution of the Purkinje cells in the cerebellar cortex is indeed included in our study. Specifically, our analysis of the spatial arrangement of the excitability features, which is reported in Fig. 8 in the revised manuscript, relies on an assessment of the spatial distribution of Purkinje cells across the cerebellar cortex and includes a meticulous reconstruction of the folia both in the target lobe and the contralateral lobe.

Moreover, we investigated whether the resolution of the segmentation process could significantly alter our findings. Hence, as described in the *Supplementary Information* document, we considered a sample MRI-based reconstructed cerebellum model from (Sereno et al., 2020), which includes a high-resolution reconstruction of the cerebellar folia, and we applied our segmentation process to this model. As shown in Fig. S5 in the *Supplementary Information* of our submission, the distribution of the orientations of Purkinje cells was similar with our segmentation process and the high-resolution segmentation provided in (Sereno et al., 2020), even though the average voxel dimension was 190μm in (Sereno et al., 2020) and 600μm in our approach. This indicates that, even though an increment in segmentation resolution may enhance the assessment of the spatial

arrangement of Purkinje cells through a finer reconstruction of the folia, the distribution of excitability features across the cerebellar cortices would not alter significantly.

Secondly, we used the quasi-uniform approximation (Bikson et al., 2013) in studying the effects of electric fields on membrane polarization and synaptic response of individual neurons. This approximation was used under the assumption that the electric field (EF) intensity may be uniform on the scale of the neurons' membrane length constant, which is realistic given that the length constant of Purkinje cells, granule cells, and deep cerebellum neurons.

Moreover, we investigated the impact that relaxing the quasi-uniform approximation would have on our results. Specifically, we considered the volume surrounding the Purkinje cell (PC) model, which is the model with the largest size and longest axon (i.e., ~700μm) in our study. Depending on the position of the Purkinje cell in the segmented volume, the cell volume can span across up to 8 voxels in our current segmentation in ROAST. We consider an 8-voxel-volume around the PC model (i.e., volume: 2.4mm × 1.2mm × 1.2mm), and we retained the field intensities $E_1, E_2, \dots, E_8$ of a randomly chosen block of 8 voxels in our ROAST segmentation. Then, we segmented the 8-voxel-volume around the PC model again by using 1μm^3-voxels, which resulted in 3.456×10^9 voxels, and we computed the voltage at every 1 μm^3-voxel by interpolating the original field values $E_1, E_2, \dots, E_8$ (distance-weighted interpolation). In this way, we ensured that different PC model compartments were in different voxels, and each compartment was polarized by the EF assigned to its own voxel. As a result, the EF was nonuniform across the neuron compartments, as reported in **Figure R2**.

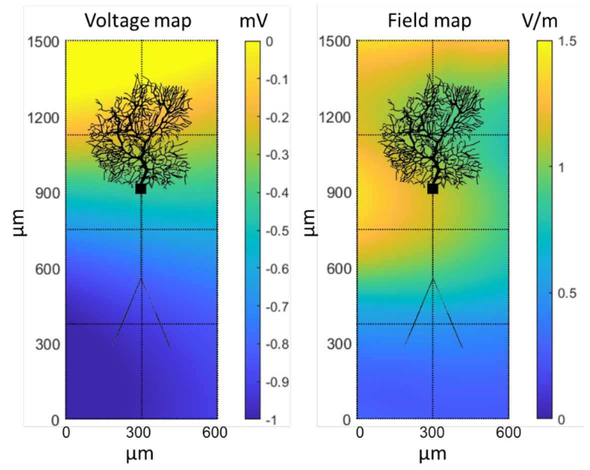

Figure R2. Colormaps reporting the voltages (*left*) and electric field intensities (*right*), respectively, across a 2,400×1,200 grid of 1-μm^3-voxels containing the Purkinje cell soma. Color scales are on the right side of each map.

We studied the effects of this nonuniform EF distribution on the PC mean firing rate under synaptic stimuli, as this metric was among the most sensitive to the applied EF. Specifically, we considered one sequence of presynaptic stimuli and correspondingly computed the FR-E curve of the PC model. The FR_E curve reports the firing rate versus the EF intensity for synaptic gain δ_{syn} =0 (i.e., same setup as in Fig. 5D, panel (*iii*), in the revised manuscript). Then, we repeated the simulations under the nonuniform EF reported in **Figure R2**.

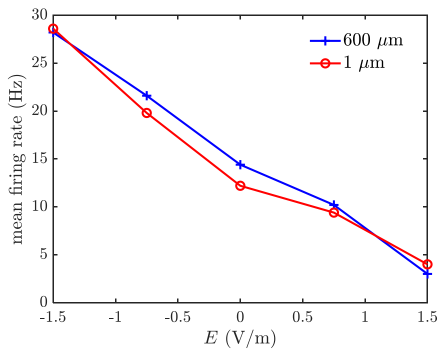

Figure R3. Mean firing rate (FR) versus electric field intensity (E) for the Purkinje cell model under synaptic inputs (δ_{syn} =0) and EF. The FR-E curve was estimated for the segmentation used in the main manuscript (voxel dimension = 600μm, *blue line*) and the high-resolution segmentation in Figure R2 (voxel dimension = 1μm, *red line*).

Figure R3 compares the FR-E curve for our original segmentation under quasi-uniform approximation (voxel dimension = 600μm) and the refined segmentation process considered in **Figure R2** (i.e., voxel dimension = 1μm).

We found that the quasi-uniform EF approximation led to a modest overestimation of the average firing rate of the

Purkinje cell throughout the range of EF intensities considered in our study. Moreover, even though the quasi-uniform approximation resulted in an underestimation of the FR-E slope, the trend of the firing rate was similar across the two segmentations as the field intensity increased. Moreover, we measured the difference between the PC silence period under EF intensities of ±1 V/m for the two segmentations. We found that, under -1-V/m-EF and +1-V/m-EF, the coarser segmentation (i.e., voxel dimension = 600μm) resulted in 0.25-ms-overestimation and 3.88-ms-understimation of the silence period, respectively, compared to the values obtained for the refined segmentation (i.e., voxel dimension = 1μm). These values correspond to an estimation error of less than 0.1% (-1-V/m-EF) and approximately 0.62% (+1-V/m-EF) of the silence period estimated for the refined segmentation.

Altogether, these results indicate the validity of the quasi-uniform approximation in our study.

Major comments:

1) The authors mentioned higher EF intensity found under the source electrode. However, there is a lack of explanation of why EF intensity in cerebellar nuclei is higher than that in the cerebellar cortex. Higher EF is observed with a greater distance between electrodes (Parazzini et al 2012, Shahid et al 2014).

Authors: The highest EF intensity occurred along the cortical tissue right under the source electrode and up to a depth of 3 mm. The average EF intensity was 2.98 ± 1.73 V/m under the electrode (max intensity: 16.56 V/m) and 0.34 ± 0.18 V/m along the cortex not covered by the electrode (mean ± S.D.). The area under the source electrode, though, was small and shallow compared to the remaining cortical tissue, thus providing a marginal impact on the sample distribution of the EF intensity across the cerebellar cortex. This explains why, despite the highest EF intensities being under the electrode, the average EF intensity across the cortices was small, and the bulk of EF intensity values remain under 0.6 V/m, as shown in Fig. 1-2 in the revised manuscript.

Furthermore, as shown in Fig. 2 in the revised manuscript, we reported a narrower sample distribution and a slightly higher average value of the EF intensity in the cerebellar nuclei compared to the cerebellar cortices. This is likely due to the nonuniform cytoarchitecture of the cerebellum. Specifically, the segmentation process of the brain scans resulted in a nonlinear distribution of the tissue conductivity across the cerebellar volume, with the segments covering the cerebellar nuclei being assigned a higher conductivity compared to the segments covering the granular layer as proposed in (Huang et al., 2013). This reflects the denser concentration of gray matter in the cerebellar nuclei compared to the cerebellar cortices and the limited density of neurons in the granular layer (Ashida et al., 2018). Moreover, this layer-specific assignment of the tissue conductivity differs from the approach in earlier work (Parazzini et al., 2012; Shahid et al., 2014), which provided a single conductivity value to all cerebellar segments. Hence, it is plausible that the presence of a continuous, thick layer of cerebellar matter with constant conductivity can explain the linear relationship between inter-electrode distance and EF intensity reported in (Parazzini et al., 2012; Shahid et al., 2014). Finally, please note that the use of different conductivity values throughout the cerebellar segments was also included in later work (Parazzini et al., 2014; Fiocchi et al., 2017).

The conductivity values assigned to the various tissue segments in our study are now reported in Table S1 in the *Supplementary Information* of the revised document.

2) Pad and ring electrode shows distinctive EF distribution; thus, it is better to include a supplementary figure1 in another figure, i.e., not a supplementary figure.

Authors: We thank the reviewer for this recommendation. The EF distributions for both types of electrodes are now reported in the main manuscript. Please, refer to Fig. 1 in the revised manuscript.

3) Insufficient explanation of why GrC and DCN were not affected by tDCS as much as PC.

Authors: We have carefully revised the morphology of the granule cell (GrC) model. We have also expanded our analysis of the response of GrC and deep cerebellar neuron (DCN) to tDCS-induced EF.

Regarding the GrC, we developed a new computational model with a more complex morphology, and we investigated the response of this GrC model to synaptic inputs under EF. We followed the approach proposed in (Masoli et al., 2020) to quantify the response to synaptic inputs from mossy fibers, and we found that EF up to 1.5 V/m had a mild impact on the postsynaptic activity of the granule cells. This is likely due to the small dimensions and deeper location of the granule cells in the cerebellum, as we observed that the EF induced a negligible variation to the GrC membrane polarization. For details about the changes to the GrC model, please, refer to our reply to point 1) on page 1 of this rebuttal. The findings of our study involving the new GrC model are now summarized in the subsection "*GrC responses to mossy fiber activation are robust against tDCS*", line 268-304 in the revised manuscript.

Regarding the DCN, we observed that the EF intensity in the cerebellar nuclei was, on average, slightly higher than in the cerebellar cortex, e.g., see sample distributions in Fig. 2A in the revised manuscript. Also, EF induced a membrane polarization of the DCN model that was comparable to the polarization of the Purkinje cells. Despite this, EF evoked negligible variations to the average firing rate of the DCN model at rest. Also, EF evoked minor changes to the shape of the bursts of action potentials that the DCN model fires in response to GABAergic inputs from Purkinje cells.

The lack of changes to the firing rate likely depends on the origins of the DCN activity at rest. (Steuber et al., 2011) reported that the spiking rate of a DCN reflects the balance between the rates at which glutamatergic and GABAergic synapses on the dendrites are activated. Since we kept the presynaptic input rates in our model at the same value with and without EF, we expected that the firing rate of the DCN model would remain largely unaffected by EF. Please, note that we kept the synaptic inputs constant throughout the application of EF for every neuron model in our study, including DCN, because our study is focused on quantifying the impact of membrane polarization on the spiking dynamics of cerebellar neurons. Previous studies, e.g., (Fritsch et al., 2010; Kronberg et al., 2017), have shown *in vitro* that EF may facilitate the long-term potentiation of synapses on pyramidal neurons, and (Rahman et al., 2013) has shown that EF can modulate the efficacy of synaptic transmission by polarizing the presynaptic axon terminals. Hence, even though the membrane polarization did not significantly alter the firing rate of the DCN model at rest, it is plausible that the firing pattern of deep cerebellar neurons will change under tDCS because of the polarization effects induced on the Purkinje cells, which directly project onto the deep cerebellar neurons. This explanation is now extensively presented in the revised manuscript. Please, see line 263-267.

The modest effect of applied EF on the after-hyperpolarization spiking of the DCN model is

related to the ionic mechanisms that mediate the post-synaptic rebound. As shown in (Steuber et al., 2011), rebound firing following strong Purkinje cell inputs requires that the chloride reversal potential in the DCN compartments is more negative than −70 mV as well as the activation of potassium voltage-gated HCN currents. In our simulations, the modest changes in membrane polarization resulted in a limited shift to the chloride reverse potential and the delay in HCN current activation, which explain the modest changes to the after-hyperpolarization rebound. This explanation is now extensively presented in the revised manuscript at line 256-259.

4) Results section:

a. Cerebellar tDCS induces mild electric fields across the cerebellum part.

Authors: Thank you. We have edited the title of the subsection.

b. P.8 177-9. Please cite previous studies and mention consistency with a previous study's result of electric field distribution of HD-tDCS.

Authors: Our results of EF distribution under HD-tDCS are consistent with findings in (Fiocchi et al., 2017), which showed that the EF evoked through HD electrodes for 1.5 mA currents remains well below 2 V/m. The major difference between the setup used in our study and (Fiocchi et al., 2017) is the montage, with Fiocchi *et al*. using two HD electrodes instead of four HD electrodes in the cerebellar area. In addition, (Fiocchi et al., 2017) used pad-shaped electrodes (radius: 6 mm; thickness: 1.3 mm; gel thickness: 6 mm) whereas we simulated ring-shaped electrodes (outer radius: 6 mm; inner radius: 4 mm). Despite these differences, though, both studies reported that the evoked electric fields in the target area are below 2 V/m. We refer to this study now in our revised manuscript on line 189.

c. Fig. 1 B (ii): Why only GrC's morphology is different from other neurons? It is better to give unity to all neuronal models' figures.

Authors: The morphology of the granule cell has been modified to reflect a more complex 3D geometry. Please, refer to our rebuttal to item 1) on page 1 of this rebuttal and to subsection "*Cerebellar neuron models*" in the revised manuscript. Also, please note that the figures reporting the cell morphology of the DCN, GrC, and Purkinje cell models have been updated to provide a uniform graphical style. Please, refer to Fig. 3A in the revised manuscript.

d. For validation of the neuron model, a linear increase in membrane polarization with the field intensity is important. However, it is hard to capture a linear increase in polarization through Table1 and explanation in the result and discussion part. It is better to use a plot or show increasing values in the table.

Authors: We agree with the reviewer. A plot has been added showing the relationship between membrane polarization and field intensity for every neuron model in our study. The plot is in Fig. 3B, panel (iii) in the revised manuscript.

5) Methods section:

   a. Please provide a specific method used to calculate EF in ROAST.

Authors: We used the following pipeline in ROAST to calculate the electric field generated by the transcranial current stimuli:

- The brain image sets were segmented into six tissue categories, i.e., white matter, gray matter, corticospinal fluid, skull, scalp, and air cavities. Each category was assigned a different conductivity value, with conductivities being reported in the *Supplementary Information* section of the revised manuscript.
- The volumetric mesh of the reconstructed 3D brain was obtained by using the tetrahedral meshing procedure proposed in (Qianqian and Boas, 2009) and implemented by the MATLAB function `iso2mesh()`.
- Finally, the Laplacian equation $\nabla^2 v = 0$, where v is the electric potential, was solved at the nodes of the volumetric mesh by using the open-source solver getDP (Dular et al., 1998) with boundary conditions given by the current intensity emitted at the anode and cathode.

This pipeline is now presented in the revised manuscript, see line 739-747.

   b. Please address information about head models (for example in Fig. 1A).

Authors: Thank you. We have now replaced the original head model (MNI152) in Fig. 1 with one of the subject-specific MRI models, i.e., atlas 5, that was used in our study. This atlas is also used in Fig. 8 in the revised manuscript. The caption has now been modified accordingly.

   c. A definition and method of polarization length and sensitivity are omitted. Please describe it with a citation.

Authors: Please, see our reply to the comment of Reviewer 1 regarding line 185. A definition of the polarization length is now reported on line 222-223 in the revised manuscript.

   d. Please provide a specific method of how-to pair (i.e., couple) EF with multi-compartmental neuronal models.

Authors: Thank you. Additional text explaining the combination of EF and multicompartment models has been included on line 192-213 in the revised manuscript.

   e. Please provide a summary of how to calculate extracellular potential, instead of just citing [79].

Authors: Thank you. We have added a summary. Please, refer to line 208-213 in the revised manuscript.

   f. If the authors used the 'extracellular mechanism' of NEURON, calculating potential is required, but the way of calculating potential was omitted. Please address it.

Authors: The reviewer is correct, i.e., we used indeed the `extracellular()` mechanism in NEURON. Please, refer to line 208-213 in the revised manuscript.

Minor comments:

- In Fig. 4A, burst period, burst interval and spiking period need to be defined with mathematical term to eliminate ambiguity.

Authors: The definitions of burst period, burst interval, and spiking period are now defined mathematically in the "*Materials and Methods*" section of the revised manuscript, on line 892-899. Briefly, we used the following definitions:

- The "*burst period*" associated with burst *k*, with *k*=1, 2, 3, …, is defined as the interval between the last spike of burst *k* and the last spike of the following burst, i.e., burst *k*+1.
- The "*burst interval*" associated with burst *k*, with *k*=1, 2, 3, …, is defined as the interval between the last spike of burst *k* and the first spike of the following burst, i.e., burst *k*+1. Hence, the burst interval is the interval between the end of a burst and the beginning of the following one.
- The "*spiking period*" of a burst is the interval between the first spike and the last spike of the burst. In our analyses, a burst is a sequence of action potentials with less than 10ms between any two consecutive action potentials.

- In Fig. 5A, the intention of the arrangement of the figures is not clear.

Authors: We have carefully revised this figure. Please, refer to the new Fig. 8 in the revised manuscript. Thank you.

Reviewer #3:

In this timely study the authors use MRI-derived reconstruction of cerebellum to 1) calculate field intensities across the cerebellum and 2) quantify the immediate impact of cerebellar tDCS on three different types of cerebellar neurons by using multi-compartments models of this neurons. The manuscript is very well written, and the figures are clear and very informative. The authors focused on the impact of tDCS on different neuronal parameters, as PC firing, complex spike wavelets or post-CS silence with important physiological consequences. I appreciate that authors clearly state the model's prediction on each one of the different sections.

Authors: We thank the reviewer for the assessment of our study.

Major comments:

1) Although authors clearly state that they only focus on PC, DCN and GrC neurons, this last group of cells (GrC) are almost missed along result sections. Considering its relevant role in the cerebellar network activity and its very characteristic morphology (ascending axon and parallel fibers) it is expected that mild changes could have strong functional impact in cerebellar processing. Although silent at rest, could exogenous electric field modulate its firing rate during sensory activation for example?

Authors: We thank the reviewer for rising this excellent point. As discussed on page 1 of this

rebuttal, we have addressed this point by carefully revising the geometry of the granule cell model and investigating the response of granule cells to synaptic stimuli under tDCS. Please, refer to our presentation in the subsection "*GrC responses to mossy fiber activation are robust against tDCS*" in the revised manuscript. Details about the revised neuron model are also provided in the subsection "*Cerebellar neuron models*" in the revised manuscript.

2) tDCS has been shown to modulate presynaptic activity *in vitro* and in alert animals. How could these presynaptic effects modify your model predictions?

Authors: We agree that the tDCS-induced modulation of presynaptic activity could percolate and affect our model predictions. This would be likely the case for the deep cerebellar neurons, which receive direct projections from the Purkinje cells, whose high sensitivity to tDCS-induced electric fields (EF) has been predicted in our study. Although plausible, it appears less likely, instead, that tDCS-induced EF would modulate the activity of both climbing fibers and mossy fibers, which are responsible for the presynaptic activity towards Purkinje cells and granule cells, respectively, mostly because of the deeper location and lack of dendritic arborization of these fibers.

As well, the parallel fibers, which are in the molecular layer and provide diffused glutamatergic synapses onto the Purkinje cells, are mostly arranged perpendicularly to the direction of the electric fields. Hence, although possible, it is unlikely that tDCS-induced EF would induce sizable changes to the presynaptic excitation provided by the parallel fibers to the Purkinje cells.

Nonetheless, we now acknowledge the possibility that tDCS could indirectly affect neurons by modulating their presynaptic activity in the manuscript. We also discuss the modeling limitations of our study in the "*Discussion*" section. Please, refer to line 674-686 in the revised manuscript.

Minor comments:

- The parcellation of the cerebellum that authors use in this study seems to have more detail than the head model. How the authors got the information about the direction of the E-field with respect to the cortical surface? When mapping the E-field direction, can the authors guarantee they are not making any errors and assigning E-field values from the CSF (for instance) to the cerebellum parcel?

Authors: We used the ROAST computational environment (Huang et al., 2019) to integrate the MRI brain images of four individuals, the position of tDCS electrodes on the skull, and the stimulation settings. Then, we segmented the images using voxels with length of 600μm and used the finite element modeling routines in ROAST to determine the intensity and direction of the EF in each voxel. We have now expanded the description of the parcellation procedure in our paper. Please, refer to line 724-747 in the revised manuscript. We also replaced the generic head model in Fig. 1A with the output model generated by ROAST in case of one subject included in our study.

Regarding the field mapping, we used the classification provided by (Park et al., 2014) for the image sets used in our study and the classification provided by (Sereno et al., 2020) for the comparison reported in Fig. S5 in the *Supplementary Information*, respectively. In both cases, authors manually corrected to isolate the cerebellar parcel. We used these parcels and excluded any electric field value that was assigned to voxels outside these parcels.

- Regarding the methods authors use to create the head model: Did authors correct manually the segmentations? Did they check the results of the segmentation?

Authors: We used the segmentations provided in (Park et al., 2014), i.e., we relied on the manually corrected version of the MRI-based head models made available by the authors in the public database at http://imaging-genetics.camh.ca/projects/subcortical-atlases/. Before using these models, though, we reviewed the segmentations and checked that they were all correct.

REFERENCES

An L, Tang Y, Wang Q, Pei Q, Wei R, Duan H, Liu JK (2019) Coding Capacity of Purkinje Cells With Different Schemes of Morphological Reduction. Front Comput Neurosci 13:29.

Asan AS, Lang EJ, Sahin M (2020) Entrainment of cerebellar purkinje cells with directional AC electric fields in anesthetized rats. Brain Stimul 13:1548-1558.

Ashida R, Cerminara NL, Brooks J, Apps R (2018) Principles of organization of the human cerebellum: macro- and microanatomy. Handb Clin Neurol 154:45-58.

Bikson M, Dmochowski J, Rahman A (2013) The "quasi-uniform" assumption in animal and computational models of non-invasive electrical stimulation. Brain Stimul 6:704-705.

Carnevale NT, Hines ML (2006) The NEURON book. Cambridge, UK ; New York: Cambridge University Press.

Chan CY, Nicholson C (1986) Modulation by applied electric fields of Purkinje and stellate cell activity in the isolated turtle cerebellum. J Physiol 371:89-114.

Consalez GG, Goldowitz D, Casoni F, Hawkes R (2020) Origins, Development, and Compartmentation of the Granule Cells of the Cerebellum. Front Neural Circuits 14:611841.

Couto J, Linaro D, De Schutter E, Giugliano M (2015) On the firing rate dependency of the phase response curve of rat Purkinje neurons in vitro. PLoS Comput Biol 11:e1004112.

De Schutter E, Bower JM (1994) An active membrane model of the cerebellar Purkinje cell II. Simulation of synaptic responses. J Neurophysiol 71:401-419.

Diwakar S, Magistretti J, Goldfarb M, Naldi G, D'Angelo E (2009) Axonal Na+ channels ensure fast spike activation and back-propagation in cerebellar granule cells. J Neurophysiol 101:519-532.

Dular P, Geuzaine C, Henrotte F, Legros W (1998) A general environment for the treatment of discrete problems and its application to the finite element method. IEEE Transactions on Magnetics 34:3395-3398.

Fiocchi S, Chiaramello E, Gazzola V, Suttrup J, Ravazzani P, Parazzini M (2017) Assessment of the capability to target cerebellar sub-regions with high-definition transcranial direct current stimulation high-definition transcranial direct current stimulation (HD-tDCS) over the cerebellum. In: 2017 IEEE 3rd International Forum on Research and Technologies for Society and Industry (RTSI), pp 1-6.

Fritsch B, Reis J, Martinowich K, Schambra HM, Ji Y, Cohen LG, Lu B (2010) Direct current stimulation promotes BDNF-dependent synaptic plasticity: potential implications for motor learning. Neuron 66:198-204.

Harris KM, Stevens JK (1988) Dendritic spines of rat cerebellar Purkinje cells: serial electron microscopy with reference to their biophysical characteristics. The Journal of Neuroscience 8:4455-4469.

Huang Y, Datta A, Bikson M, Parra LC (2019) Realistic volumetric-approach to simulate transcranial electric stimulation-ROAST-a fully automated open-source pipeline. J Neural Eng 16:056006.

Huang Y, Dmochowski JP, Su Y, Datta A, Rorden C, Parra LC (2013) Automated MRI segmentation for individualized modeling of current flow in the human head. J Neural Eng 10:066004.

Jaeger D, Bower JM (1994) Prolonged responses in rat cerebellar Purkinje cells following activation of the granule cell layer: an intracellular in vitro and in vivo investigation. Exp Brain Res 100:200-214.

Khaliq ZM, Gouwens NW, Raman IM (2003) The contribution of resurgent sodium current to high-frequency firing in Purkinje neurons: an experimental and modeling study. J Neurosci 23:4899-4912.

Kronberg G, Bridi M, Abel T, Bikson M, Parra LC (2017) Direct Current Stimulation Modulates LTP and LTD: Activity Dependence and Dendritic Effects. Brain Stimul 10:51-58.

Lang EJ, Tang T, Suh CY, Xiao J, Kotsurovskyy Y, Blenkinsop TA, Marshall SP, Sugihara I (2014) Modulation of Purkinje cell complex spike waveform by synchrony levels in the olivocerebellar system. Front Syst Neurosci 8:210.

Legendy CR, Salcman M (1985) Bursts and recurrences of bursts in the spike trains of spontaneously active striate cortex neurons. J Neurophysiol 53:926-939.

Leto K, Carletti B, Williams IM, Magrassi L, Rossi F (2006) Different types of cerebellar GABAergic interneurons originate from a common pool of multipotent progenitor cells. J Neurosci 26:11682-11694.

Masoli S, Solinas S, D'Angelo E (2015) Action potential processing in a detailed Purkinje cell model reveals a critical role for axonal compartmentalization. Front Cell Neurosci 9:47.

Masoli S, Tognolina M, Laforenza U, Moccia F, D'Angelo E (2020) Parameter tuning differentiates granule cell subtypes enriching transmission properties at the cerebellum input stage. Commun Biol 3:222.

Mittmann W, Hausser M (2007) Linking synaptic plasticity and spike output at excitatory and inhibitory synapses onto cerebellar Purkinje cells. J Neurosci 27:5559-5570.

Pan MK, Li YS, Wong SB, Ni CL, Wang YM, Liu WC, Lu LY, Lee JC, Cortes EP, Vonsattel JG, Sun Q, Louis ED, Faust PL, Kuo SH (2020) Cerebellar oscillations driven by synaptic pruning deficits of cerebellar climbing fibers contribute to tremor pathophysiology. Sci Transl Med 12:eaay1769.

Parazzini M, Fiocchi S, Ravazzani P (2012) Electric field and current density distribution in an anatomical head model during transcranial direct current stimulation for tinnitus treatment. Bioelectromagnetics 33:476-487.

Parazzini M, Rossi E, Ferrucci R, Liorni I, Priori A, Ravazzani P (2014) Modelling the electric field and the current density generated by cerebellar transcranial DC stimulation in humans. Clin Neurophysiol 125:577-584.

Park MT, Pipitone J, Baer LH, Winterburn JL, Shah Y, Chavez S, Schira MM, Lobaugh NJ, Lerch JP, Voineskos AN, Chakravarty MM (2014) Derivation of high-resolution MRI atlases of the human cerebellum at 3T and segmentation using multiple automatically generated templates. Neuroimage 95:217-231.

Qianqian F, Boas DA (2009) Tetrahedral mesh generation from volumetric binary and grayscale images. In: 2009 IEEE International Symposium on Biomedical Imaging: From Nano to Macro, pp 1142-1145.

Radman T, Ramos RL, Brumberg JC, Bikson M (2009) Role of cortical cell type and morphology in subthreshold and suprathreshold uniform electric field stimulation in vitro. Brain Stimul 2:215-228, 228 e211-213.

Rahman A, Reato D, Arlotti M, Gasca F, Datta A, Parra LC, Bikson M (2013) Cellular effects of acute direct current stimulation: somatic and synaptic terminal effects. J Physiol 591:2563-2578.

Rezaee Z, Dutta A (2019) Cerebellar Lobules Optimal Stimulation (CLOS): A Computational Pipeline to Optimize Cerebellar Lobule-Specific Electric Field Distribution. Front Neurosci 13:266.

Robin K, Maurice N, Degos B, Deniau JM, Martinerie J, Pezard L (2009) Assessment of bursting activity and interspike intervals variability: a case study for methodological comparison. J Neurosci Methods 179:142-149.

Sereno MI, Diedrichsen J, Tachrount M, Testa-Silva G, d'Arceuil H, De Zeeuw C (2020) The human cerebellum has almost 80% of the surface area of the neocortex. Proc Natl Acad Sci U S A 117:19538-19543.

Shahid SS, Bikson M, Salman H, Wen P, Ahfock T (2014) The value and cost of complexity in predictive modelling: role of tissue anisotropic conductivity and fibre tracts in neuromodulation. J Neural Eng 11:036002.

Solinas SM, Maex R, De Schutter E (2006) Dendritic amplification of inhibitory postsynaptic potentials in a model Purkinje cell. Eur J Neurosci 23:1207-1218.

Steuber V, Schultheiss NW, Silver RA, De Schutter E, Jaeger D (2011) Determinants of synaptic integration and heterogeneity in rebound firing explored with data-driven models of deep cerebellar nucleus cells. J Comput Neurosci 30:633-658.

Svirskis G, Baginskas A, Hounsgaard J, Gutman A (1997) Electrotonic measurements by electric field-induced polarization in neurons: theory and experimental estimation. Biophys J 73:3004-3015.
